# The Outcomes of Health Education Programme on Stress Level Among the Caregivers of Post Total Knee Replacement Surgery

**DOI:** 10.3389/fpsyt.2021.571027

**Published:** 2021-03-31

**Authors:** Md. Fadlisham Samsuddin, Jalina Karim, Azizul Akram Salim

**Affiliations:** ^1^Department of Nursing, Faculty of Medicine & Health Sciences, University Malaysia Sarawak, Kota Samarahan, Malaysia; ^2^Department of Nursing, Faculty of Medicine, Universiti Kebangsaan Malaysia Medical Centre, Kuala Lumpur, Malaysia; ^3^Department of Orthopaedics, School of Medical Sciences, Universiti Sains Malaysia, Kubang Kerian, Malaysia; ^4^Hospital Universiti Sains Malaysia, Kota Bharu, Malaysia

**Keywords:** caregiver burden, stress level, health education programme, total knee replacement, mental health

## Abstract

**Introduction:** Stress level among the caregivers is often related to caregivers' lack of knowledge and skill to care for the patients. A health education program to the caregivers is one of the important elements in increasing the knowledge and skills in managing patients at home. The specific objectives of this study were to determine caregiver's stress level in managing post total knee replacement (TKR) patients pre and post of a health education program.

**Materials and Methods:** A clinical intervention trial design was conducted in Hospital Universiti Sains Malaysia (HUSM) with a sample size of 32 caregivers. A validated Zarit Burden Interview (ZBI) questionnaire was used to measure the stress level pre and post of the health education program on the management of patients post-TKR surgery which was adopted from Fresno Surgical Hospital in California.

**Results:** The findings showed that there was a significant difference between pre and post level of stress (*p* ≤ 0.001).

**Conclusion:** This study revealed the positive outcome of the health education program. It reduced the stress level among the caregivers in caring for their relatives with post-TKR surgery.

## Introduction

With the increasing number of healthcare costs, medical advances, shorter hospital stays, and limited discharge planning, many responsibilities of long-term health care have moved from the hospital setting to the home environment (1). Once the care recipient is discharged home, family members are expected to provide much care to an adult with chronic illness and/or disability, for example; patients with post TKR (1). Caregivers of patients who underwent elective knee replacement play important roles in the early recovery process due to today's short hospital stays, and the increased age of patients (2). Caregivers often experience lower levels of psychological well-being, as well as a financial and physical burden during caregiving the patients of post-TKR surgery (3). It may therefore be a challenge for them to support the patients during the recovery period in their home environment. Also, caregiving responsibilities have expanded well-beyond assisting the patients' post-TKR with traditional household chores to now include performing medical and nursing tasks provided by the medical professionals in the hospital setting (4). According to Caron et al. (5), hospital admission is a stressful ordeal and often represents only the beginning of a long rehabilitation period of which hospital discharge is an important landmark.

It is estimated between 40 and 70% of caregivers exhibit clinically significant depressive symptoms with approximately one-quarter to one-half indicates the diagnostic criteria for major depression (6). Studies have shown that the stress of caregiving has put caregivers at a higher risk for chronic health problems such as cardiovascular problems and high blood pressure with an estimated 17–35% of caregivers perceiving their health as fair to poor (7).

Total knee replacement (TKR) is rapidly becoming one of the most common elective inpatient surgeries in the United States (8). In 2003 the number of TKRs performed in the United States was 402,100 (9). This number is expected to double by the year 2015 according to growth trends, even without factoring in the growing elderly population (9). TKR is often an effective elective surgery for patients whose quality of life has decreased because of pain and functional disability resulting from osteoarthritis (10).

Informal caregiving can facilitate a positive outcome for patients (11). Caregiver experienced more burden and less self-esteem especially among the spouses, and spouses of older adults reported that they felt less informed during the discharge process than caregiving adult children. It is known that emotional support from family caregivers can improve patients' recovery outcomes after knee replacement, for example by strengthening patients' beliefs in their ability to manage recovery and providing positive emotional responses to improve the patients' recovery (12).

Results from the European project showed that caregivers and patients who underwent knee replacement expected a wide range of knowledge and those expectations were not adequately met during the hospital stay (13). Caregivers may therefore lack empowering knowledge and may not be able to experience empowerment during patients' recovery process. Patient and caregiver education is linked to and promote the recovery process, and continuous and active involvement in healthcare results in better postoperative outcomes for the patients and their caregivers (14). Patients from the Nordic countries who underwent knee replacement surgery reported that caregiver involvement in patient education should be improved to make them more satisfied with the care they received (15). Caregivers with fulfilled knowledge expectations may feel empowered to support the patients during the early recovery period, and this may reduce their stress level in caregiving and at the same time may have a positive effect on patients' quality of recovery (QoR).

Research on caregiver's support of patients who underwent knee replacement is limited, in contrast to research on other medical issues, for example, persons with heart failure, dementia, chronic illness, or diabetes (16). To our knowledge, few studies have been made on caregiver's support of patients who underwent hip or knee replacement (17). However, the stress level among caregivers with patients post-TKR has not been studied before. As patients in the Nordic sample of the main project were least satisfied with how nurses prepared their caregivers before discharged from the hospital (15), we decided to conduct further analyses on the stress level among the caregivers with patients post-TKR; pre and post-intervention of health education program related to specific care after TKR surgery. This knowledge may help nurses to identify caregivers in need of support during the period of hospitalization.

In recent years, considerable effort has been made to describe the population of caregivers and examine the positive and negative consequences of caregiving (18). Positive outcomes of caregiving include personal growth, strengthening of the relationship between caregivers and care recipients, feelings of satisfaction, and increased self-esteem (19). Negative consequences of caregiving may be physical, financial, psychological, or social (18). Specifically, these consequences may include isolation, increased responsibilities, loss of employment, depression, a decline in physical health, financial strain, feelings of burden, and stress (20). The outcomes of stress and burden are central features of most caregiving models (18). Hence, the current study aimed to determine the difference in the stress level between pre and post health education program among caregivers of post-TKR patients.

## Materials and Methods

A clinical intervention trial design was used to conduct this study. This study used “one-group pre-test and post-test design,” and “health education programme” was the independent variable which also served as an intervention in this study. This study was conducted among the caregivers of the patients who underwent TKR surgery in male and female orthopedic wards in Hospital Universiti Sains Malaysia (HUSM) Kubang Kerian, Kelantan during day one post-operation (POD1) and orthopedic clinic during the follow-up appointment.

The participants for this study were 32 caregivers of chronic knee osteoarthritis (OA) patients who were selected by using purposive sampling. Definition of caregivers are consist of patient's family members such as husband or wife, children or relatives who accompanied, consistently in delivery care, and managed the patients inward and at home, after they underwent TKR surgery and during follow-up. The sample size determination for participants in this study was by applying the formula of Krejcie and Morgan sample size calculation. Based on the calculation, the sample size of the participants supposed to be 40 participants. However, due to a few factors, the researcher obtained only 32 participants for this current study. The participants were those who accompanied, cared and managed the patients inward and at home, after they underwent TKR surgery and during TCA in the orthopedic clinic at HUSM. The participants who were selected must have the required inclusion criterion such as relatives who consistently in delivery care for the patients post-surgery, Malaysian, aged 21-year-old and above and able to understand the Malay language well while for exclusion criterion were such as having any medical problems (e.g., cancer under active treatment, diabetes, hypertension, and renal failure), frequent change of caregiver and first TCA is more than two weeks after the patients are discharged.

The intervention protocols involved in this study were the implementation of the structured health education programme (HEP) and pre and post self-administered questionnaire by Zarit Burden Interview. The health education programme used the post-TKR education package by Fresno Surgical Hospital in California. It consists of 10 elements that are; (1) pain management, (2) exercise, (3) preventing constipation, (4) preventing blood clot, (5) diet/nutrition, (6) medications, (7) surgical wound care, (8) durable medical equipment, (9) TCA, and (10) complications. The HEP was validated by two expert panels in orthopedic management. The health education programme was conducted once a day to each caregiver between 15 and 20 min on the day one of post-operation (POD1) of TKR. In conducting the HEP session, explanation to the caregivers was given in both English and Malay language. This is considering that most of the caregivers were not proficient users of English and the researcher speaks both languages fluently. Before the HEP session, the caregivers were required to answer the questionnaire (pre-test). The same questionnaire was given to the caregiver during the TCA to re-assess the outcome of the health education programme that was conducted to them before.

A validated questionnaire of Zarit Burden Interview was used as an instrument for this current study. The questionnaire consists of 22-items and each item uses a 5-point Likert scale ranging from 0 to 4; where 0 is never and 4 is always or almost always stress. The pilot study was conducted to 30 caregivers before the actual study to evaluate the proficiency of the questionnaire and intervention of the HEP. The result was 0.967 which in the range of 0.70 to 0.83 as suggested by Schrag et al. (21).

Ethical approval for this study was obtained from the Universiti Sains Malaysia Human Research Ethics Committee (JEPeM) and UKMMC Research Ethics Committee. All participants were given written consent before the session.

The data were analyzed by using SPSS version 24 for descriptive analysis such as mean, standard deviation, frequencies and percentage to outline the participants' descriptive characteristics. Categorical data such as age, gender, race, education level, marital status, employment, monthly income, and relationship of participants were analyzed using frequency and percentage while continuous data such as stress level was analyzed using mean and standard deviation. All continuous data had first been tested for its normality before proceeding to the inferential statistics.

## Results

The findings of the subjects' socio-demographic characteristics were illustrated in Table 1. Almost half of the participants fell into the 51–60-year-old age group (43.8%), while another half of them were from a different category of age with percentage, respectively. From this intervention group, there was 19 female (59.4%) caregivers and 13 males (40.6%). The data for socio-demographic characteristics in this current study were collected based on a study about caregivers' morbidity in palliative care unit: predicting by gender, age, burden and self-esteem (22).

**Table 1:** Data of socio-demographic characteristics of participants.

**Variables**	**Mean**	**Std deviation**	**Frequency** ** (*n* = 32)**	**Percentage (%)**
**Age**	51.19	12.09		
21–30			1	3.1
31–40			7	21.9
41–50			4	12.5
51–60			14	43.8
61–70			5	15.6
71–80			1	3.1
**Gender**				
Male			13	40.6
Female			19	59.4

To determine the difference in the stress level between pre and post HEP among caregivers of post TKR patients, a repeated measurement ANOVA was used. Based on the analysis of the Zarit Burden Interview (ZBI) questionnaire, there was a significant difference between the means of the pre and post-stress level of participants (*p* ≤ 0.001). The total mean score for the post-stress level was 28.03 (*sd* = 14.36), which was lower than the total mean score for a pre-stress level, 40.91 (*sd* = 19.48) with mean different was −12.87 and its 95% confidence interval of different of lower was −17.15 and upper was −8.60 as shown in Table 2.

**Table 2:** Means of pre and post-stress level of participants (*n* = 32).

**Mean (SD)**							
**Variables**	**Pre**	**Post**	**Mean differ**	**95% CI of the difference**		***t*****!!break (df)**	***p***
				**Lower**	**Upper**		
Total score of stress	40.91!!break (19.48)	28.03 (14.36)	−12.88	−17.15	−8.60	−6.15 (31)	≤0.001

*A repeated measurement ANOVA, p < 0.05*.

## Discussion

In managing patient care after surgery, most caregivers often experience stress and anxiety. The stress level increases when the caregivers lack the knowledge and appropriate skills and have no experience in patient care post-surgery. According to studies which adopted an online method of delivering psycho-educational interventions to participants, revealed a substantial improvement in caregivers' knowledge levels (22), stress, and social support levels (23). Additionally, lack of exposure to the structured and systematic discharge planning and HEP among the caregivers before the patients were discharged, contributed to the increase of caregivers' stress level. According to Sigurdardottir et al. (24), family psycho-educational programmes for patients and their caregivers were effective in improving physical and emotional health. There was evidence that psychosocial interventions improve coping, self-efficacy and reduce psychological distress among the caregivers of patients with OA (24).

Similar to this study, the level of stress among the caregivers decreased in which the results indicated a significant difference before and after the intervention. This indicated that the HEP conducted on the caregivers of patients who had undergone TKR surgery was significantly effective and the interventions showed additional positive effects on the caregivers. Sufficient health education and training among caregivers can greatly reduce anxiety, depression and stress within their caregiving duties, ultimately prolonging their ability to provide care at home (25).

Another study by Tay Swee Cheng et al. (26) revealed that primary caregiver gained benefit in terms of reducing depression, more notably, strain at 6 months. Based on their findings, intervention caregivers' mean depression scores trended downward more than controls; mean strain scores remained stable in the intervention group but trended toward an increase in the control group. The findings were amplified among caregivers who provided more than 14 h of weekly assistance at baseline, the strain at 6 months was significantly lower in the intervention group. The observed effects of the intervention were both stronger among higher intensity caregivers and consistent across two distinct outcomes suggested that observed effects were due to the intervention (26).

Furthermore, a study by Wang et al. (27) mentioned that a greater reduction in caregiver stress scores baseline to post-intervention was attributed to their participation in the health education intervention. They might have gained new caregiving skills in coping with patient care post-surgery. Besides, they might have gained more confidence to deal with their relative's care during at home. This is consistent with earlier studies about the positive effects of psycho-education interventions on family burden (27). In this current study, it is found that HEP plays the main role and an important element in reducing stress level among the caregivers and also increases their knowledge and skills in patient care post-TKR surgery.

This study revealed that the sample size of the total population was too small (*n* = 32). This is because it was conducted with the participants of osteoarthritis patients from one hospital only which was Hospital Universiti Sains Malaysia (HUSM). Thus, this factor was the limitation of the current study.

It is essential to equip the caregivers with knowledge on TKR and its management especially when caregivers have limited formal education. This is because an increase in knowledge about patients' care post-TKR surgery might help reduce the caregivers' sense of burden and reduce their stress level. Further, information regarding the patients' care after TKR surgery, as well as potential complications facing the patient post the operation, should be explicitly explained to the caregivers. Hence it can reduce reliance on hospital services. Thus, more research is needed to explore the concept of optimal HEP for caregivers. This research could be extended to experimental study to investigate the effectiveness of HEP with the control and intervention group.

This study highlights the caregivers can be the helpful resources in the management of patients post-TKR. More consideration needs to be given to caregivers' of the patient, such as considering caregivers' health knowledge in the development of a health education programme. More information must be provided to those who live with knee osteoarthritis patients; caregivers' knowledge should be improved, especially for the management of patients post-TKR, for which they depend on the help of others. With information delivering as a major factor in perceived health-related competence, caregivers should be informed about their important role in managing patients post-TKR. Information about medications, exercises or physiotherapy, appointment, symptoms, diet and supportive measures could enhance their motivation and behavioral skills in such situations. Providing caregivers of patients post-TKR with such information could improve the quality of recovery; however, further studies are needed to confirm these findings and to explore health education programme in managing patients post-operative for other chronic bone diseases.

## Conclusion

This study emphasized the importance and outcome of HEP for the caregivers of post TKR patients. More understanding of these specific caregiver needs and concerns by nurses could play an important role in developing caregiver centered care of patients with post-TKR surgery. Meanwhile, the facilitated application of the health education programme to the caregivers in caring for relatives who underwent TKR surgery provided a way of identifying their educational needs. It thus facilitated in examining and comparing the patterns of health needs in caregiver education. The design and findings of this study provided useful information on needs assessment and subsequent design of appropriate education programmes by nurses and healthcare professionals when providing caregiver-centered care in community settings.

Furthermore, based on findings of this current study it showed that stress level among the caregivers decreased after the intervention of HEP. This had proven that the HEP conducted on the caregivers of patients who had undergone TKR surgery was significantly effective and the intervention might have additional positive effects on the caregivers. This indicated that providing caregivers with health education and training can greatly reduce anxiety, depression and stress within their caregiving duties, ultimately prolonging their ability to provide care at home.

## Data Availability Statement

The raw data supporting the conclusions of this article will be made available by the authors, without undue reservation.

## Ethics Statement

The studies involving human participants were reviewed and approved by Universiti Sains Malaysia Human Research Ethics Committee and Research and Ethics Committee of Universiti Kebangsaan Malaysia Medical Center. The patients/participants provided their written informed consent to participate in this study.

## Author Contributions

MS: data collection, data analysis, and manuscript writing. JK: conceptual framework and manuscript editing. AS: conceptual framework and manuscript editing. All authors contributed to the article and approved the submitted version.

## Conflict of Interest

The authors declare that the research was conducted in the absence of any commercial or financial relationships that could be construed as a potential conflict of interest.
